# Juvenile erythrocytosis in children after liver transplantation: prevalence, risk factors and outcome

**DOI:** 10.1038/s41598-020-66586-6

**Published:** 2020-06-16

**Authors:** Maddalena Casale, Domenico Roberti, Claudia Mandato, Raffaele Iorio, Maria Caropreso, Saverio Scianguetta, Stefania Picariello, Silverio Perrotta, Pietro Vajro

**Affiliations:** 1 Department of Woman, Child and General and Specialized Surgery, University of Campania Luigi Vanvitelli, Naples, Italy; 2 Department of Pediatrics, Children’s Hospital Santobono-Pausilipon, Naples, Italy; 3 Department of Translational Medical Science, Section of Pediatrics, University of Naples Federico II, Naples, Italy; 4 Department of Pediatrics, Buon Consiglio Fatebenefratelli Hospital, Naples, Italy; 5 Pediatric Clinic, Department of Medicine, Surgery and Dentistry, University of Salerno, “Scuola Medica Salernitana”, Baronissi (Salerno), Italy

**Keywords:** Paediatric research, Genetic testing

## Abstract

Most reports of post-transplant erythrocytosis have involved kidney recipients and, so far, there have been no large studies of onset of erythrocytosis after orthotopic liver transplantation (OLT) in children. We present a long-term survey of pediatric liver recipients, evaluating prevalence, outcome and the main potential causes of erythrocytosis, including a comprehensive mutational analysis of commonly related genes (mutations of *HBB* and *HBA*, *JAK2*, *EPOR*, *VHL*, *EPAS1* and *EGLN1*). Between 2000 and 2015, 90 pediatric OLT recipients were observed for a median period of 8.7 years (range 1–20.4 [IQR 4.9–13.6] years). Five percent of the study population (4 males and 1 female) developed erythrocytosis at 8.5 years post OLT (range 4.1–14.9 [IQR 4.7–14.7]) at a median age of 16.6 years (range 8.2–18.8 [IQR 11.7–17.7]). Erythrocytosis-free survival after OLT was 98.6% at 5 years, 95% at 10 years, and 85% at 15 years, with an incidence rate of 6/1000 person-years. No cardiovascular events or thrombosis were reported. No germinal mutation could be clearly related to the development of erythrocytosis. One patient, with high erythropoietin levels and acquired multiple bilateral renal cysts, developed clinical hyper-viscosity symptoms, and was treated with serial phlebotomies. In conclusion, this prospective longitudinal study showed that erythrocytosis is a rare complication occurring several years after OLT, typically during adolescence. Erythrocytosis was non-progressive and manageable. Its pathogenesis is still not completely understood, although male gender, pubertal age, and renal cysts probably play a role.

## Introduction

Erythrocytosis is defined by an increase in red-cell mass to >125% of the predicted valued according to sex and body mass^1^. In adults, hematocrit (Hct) >56% in females and 60% in males has been considered sufficient to define the condition of absolute erythrocytosis^2^, although the World Health Organization has now proposed lower levels among the criteria for Polycythemia Vera (i.e. Hct > 49% or >48% and Hemoglobin [Hb] > 16.5 g/dL or >16 g/dL, respectively, for males and females)^3^. In children, diagnosis can be challenging as it relies on Hb and Hct percentiles and cut-off values that vary according to age and sex, even though fixed cut off criteria are used for adults. Furthermore, some diagnostic tests for erythrocytosis may require specific procedures for use in pediatric populations, making them more complex and expensive than those used in adults^4^.

The main concern related to erythrocytosis in the general population, and especially in patients undergoing organ transplantation, is the risk for cardiovascular events and death, as erythrocytosis can affect transplant outcome and lead to long-term complications^5^.

Erythrocytosis is quite a common complication after renal transplants with a prevalence of 10–15%^5^, varying from as low as 3.2% in children^6^ to as high as 20.2% in adults^5–7^. Erythropoietin (EPO), renin-angiotensin system, insulin-like growth factor, male gender and renal cysts are some of the factors that seem to play a role in post-renal transplant erythrocytosis^5,7–10^. Simultaneous kidney-pancreas transplantation seems to increase the risk for the development of post-transplant erythrocytosis as compared to kidney transplant only^11^. Diagnosis and treatment of post-transplant erythrocytosis (PTE) are important to maintain Hct below a critical threshold in order to reduce clinical symptoms and to minimize the possible risk of hyperviscosity-related thrombosis, especially at vascular anastomosis sites^5,8,12^. In kidney-transplant recipients, post-transplant erythrocytosis (PTE), as in other forms of erythrocytosis, is often symptomatic with malaise, headache, plethora, lethargy, and dizziness. Thromboembolic events often occur (10–30%), eventually leading to death. These events can involve both veins and arteries, and present as thrombosis of digital or branchial arteries, thrombophlebitis, stroke, or pulmonary embolus^5^.

Prevalence and causes of erythrocytosis were studied in a cohort of 96 adults in follow up for at least one year post-orthotopic liver transplantation (OLT)^13^. Excluding secondary forms, idiopathic erythrocytosis was observed in 11 out of 96 (11%). Male sex, history of HBV infection, and hepatitis B immune globulin therapy were suggested to be possible co-factors for risk of erythrocytosis. Patients required serial phlebotomy to maintain Hct level between 45% and 49%, and no cardiovascular events were reported during an 18-month follow up. However, the Authors reported cardiovascular events in two patients several years after OLT.

Unfortunately, there are no reliable long-term data on prevalence, causes, treatment and outcome of post-OLT erythrocytosis in children. In a small pediatric cohort, 3 out of 10 patients required phlebotomy 5–9 days after liver transplantation due to a progressive and persistent increase in Hb levels^14^. However, the study was compromised by short follow up and the limited sample size.

Here, in this prospective longitudinal study, we aimed to determine the prevalence, causes and outcomes of post-OLT erythrocytosis in a large cohort of children with long-term follow up in order to provide additional information regarding its natural course.

## Methods

Ninety consecutive patients followed for OLT at the Pediatric Departments of University Federico II in Naples and University Hospital in Salerno between 2000 and 2015 were assessed for the occurrence of erythrocytosis. Procedures and genetic analysis for a diagnosis of erythrocytosis were performed at the Pediatric Department of the University of Campania “Luigi Vanvitelli” in Naples.

Parents and/or legal guardians gave their informed consent for the study, which was carried out in agreement with the Declaration of Helsinki of 1975, as revised in 2008. The study was approved by the Ethics Committees of the University of Campania “Luigi Vanvitelli” in Naples.

Patients’ family and personal history, complete physical examination, symptoms possibly related to erythrocytosis (e.g. headache, malaise, dizziness, arterial hypertension), red blood cell count, Hb levels, Hct, calcineurin inhibitor blood levels, hepatic and renal function tests, including abdominal ultrasound parameters, were obtained from patients’ files.

Criteria for diagnosis of erythrocytosis were:

- persistent Hb or Hct greater than 97^th^ percentile of method-specific reference range for age, sex, and altitude of residence with normal white blood cell and platelet counts^15,16^;
- spleen dimension within normal values for age and sex^17^.

**Figure 1:**
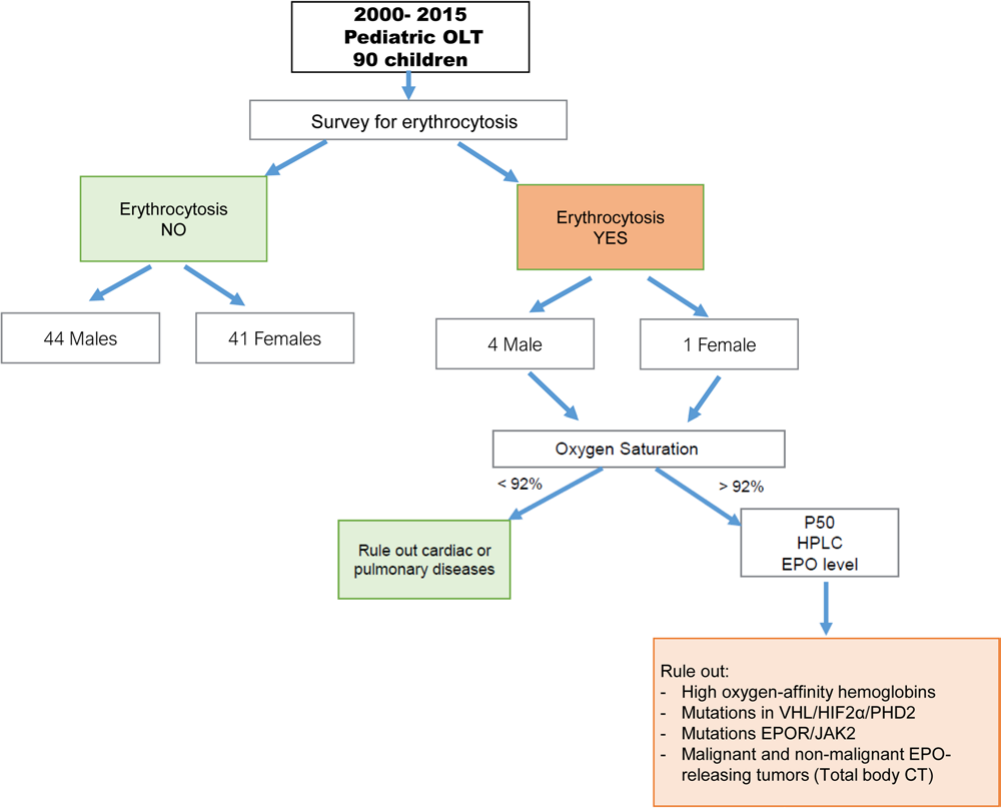
Study protocol and procedures. OLT: orthotopic liver transplantation; P50: partial pressure of oxygen required to achieve 50% saturation of hemoglobin binding sites; EPO: erythropoietin; VHL: Von Hippel-Lindau; HIF2α: Hypoxia Inducible Factor 2 alpha; PHD2: Prolyl hydroxylase domain protein 2; EPOR: Erythropoietin Receptor; JAK2: Janus kinase 2; CT: computed tomography.

As per study protocol (Fig. 1), all children with a diagnosis of erythrocytosis underwent intermittent pulse oximetry to assess oxygen saturation, blood gas analysis to assess P50 (i.e. the partial pressure of oxygen required to achieve 50% saturation of Hb binding sites), high performance liquid chromatography to detect high oxygen-affinity hemoglobins (defect in α and β globin genes), and EPO quantification.

In cases in which secondary erythrocytosis was excluded, mutations of α and β globins, Janus kinase 2 (*JAK2 V617F* and *exon 12 mutations*), erythropoietin receptor (EPOR), Von Hippel-Lindau (VHL), hypoxia inducible factor 2α (HIF2α) and prolyl hydroxylase domain protein 2 (PHD2) genes were investigated. Genomic DNA was isolated using a Flexigene DNA purification kit (Qiagen, Lane Valencia, CA, USA). The exons and the exon-intron boundaries were amplified by polymerase chain reaction (PCR). The ABI 310 DNA Sequencer and the ABI PRISM Dye Terminator Cycle Sequencing Reaction Kit (Applied Biosystems, Milan, Italy) were used according to the manufacturer’s instructions^18–23^. To analyze VHL transcript variation in Patient 2, total RNA from the patient’s peripheral blood leukocytes was retro-transcribed and cDNA was amplified using primers localized in 5′-and 3′-UTR of VHL mRNA.

Renin serum levels were evaluated with ELISA (IBL International GMB H, Germany).

Central hypoxia-driven processes due to smoking habit, high altitude, chronic lung disease, sleep apnea, and congenital cyanotic heart disease were investigated through evaluation of patients’ personal history and chest X-ray, along with spirometry, otorhinolaryngologic evaluation and echocardiogram.

Patients with high EPO levels underwent total body computed tomography to exclude malignant and non-malignant tumors associated with increased EPO production.

### Statistical analysis

Statistical analysis was performed with SPSS 23 software for Windows. Continuous variables and categorical variables were expressed as median (range and interquartile range [IQR]) and percentage, respectively. Comparisons were made using Mann-Whitney U test for continuous non-parametric variables and Fisher Exact test for categorical variables. p < 0.05 was considered statistically significant. Kaplan-Meier analysis was used to estimate erythrocytosis event-free survival (EFS). Erythrocytosis incidence rate was calculated as the number of new cases per person-year. Wilkoxon signed-rank test was performed for non-parametric continuous variables to investigate differences between paired observations. In particular, we assessed differences between pre- and post-liver transplantation Hb and Hct in patients with PTE.

## Results

### Patients’ demographics

Ninety pediatric liver recipients (42 female and 48 male) were followed for a median period of 8.7 years (range 1–20.4 [IQR 4.9–13.6]). Median age at liver transplantation was 1.5 years (range 0.2–16.5 [IQR 0.8–4.1]). Main OLT indication was biliary atresia (63 out of 90; 70%). Other indications were cryptogenetic cirrhosis (n = 4), progressive familial intrahepatic cholestasis (n = 3), fulminant hepatic failure (n = 3), Crigler Najjar type 1 (n = 2), Alagille syndrome (n = 2), autoimmune hepatitis (n = 2), hepatic tumor (n = 2), other (n = 9).

Family history was not informative and parents’ complete blood counts were normal. None of the patients had a smoking habit or lived at high altitude.

Physical examination of the patients showed no relevant abnormalities.

Biochemical evaluation to assess function of other principal body organs and systems was normal. EPO levels were normal in all patients except one. Routine echocardiograms confirmed that none of the patients was affected by a congenital cyanotic heart disease. Chest X-rays and spirometry results were within normal limits in all patients. Repeated blood gas analyses and P50 value assessment were also normal in all patients.

### Post-transplant treatment

Post-transplant primary immunosuppressors were tacrolimus in 64 (71%) and cyclosporin A in 26 (29%) patients. All patients had been on immunosuppressive treatment since OLT, with drug level monitoring at intervals of 1–3 months to maintain the therapeutic level and avoid toxicity. None of the 90 children had pre-OLT erythrocytosis, and no renal cysts were found at routine abdominal ultrasound (US) or computed tomography (CT) scans performed before OLT. None of the study patients presented with clinical or laboratory signs of hepatopulmonary syndrome, according to previously described criteria^24^. None of the patients had HBV or received hepatitis B immune globulin therapy.

### Erythrocytosis diagnosis and analysis

During follow up, erythrocytosis was diagnosed in 5 patients (5.5%) at a median age of 16.6 years (range 8.2–18.8 [IQR 11.7–17.7]). There was a statistically significant median increase in Hct (14.9%, IQR 14.2–19.6, range 14–20.2) from pre-liver transplant values (35%, IQR 32–37.1, range 31.1–37.2) to post-liver transplant values (51.1%, IQR 51.5–52.5, range 49–53, p < 0.05). Similarly, Hb showed a median increase of 5.8 g/dl (IQR 4.7–7.4, range 4.3–7.5) from pre-transplant values (12 g/dl, IQR 10.4–12.3, range 10.2–12.6) to post-transplant values (17.7 g/dl, IQR 17–17.9, range 16.3–18, p < 0.05) (Table 1). Median time from transplantation to diagnosis of erythrocytosis was 8.5 years (range 4.1–14.9 [IQR 4.7–14.7]), with an incidence rate of 6/1000 person-years. Four out of 5 patients were male. There was no significant difference in age at OLT, duration of follow up, or age at last assessment between patients with and those without a post-transplant diagnosis of erythrocytosis (Table 2).

**Table 1:** Demographic, clinical and laboratory findings of five children and adolescents who developed post-liver transplantation erythrocytosis.

Patient	1	2	3	4	5
Gender	M	M	M	F	M
OLT indication	Crigler Najjar I	Biliary Atresia	Biliary Atresia	Biliary Atresia	Biliary Atresia
OLT Age (years)	10.3	12.5	1.8	2.9	0.8
Age at PTE diagnosis (years)	18.8	16.6	16.7	8.2	15.3
Immunosuppressor at PTE diagnosis	Tacrolimus	Tacrolimus	Tacrolimus	Tacrolimus	Cyclosporin A
Hct pre-OLT (%)	37.2	37.1	31.1	35	32.8
Hct at PTE diagnosis (%)	51.5	52	50	49	53
Hct at last control (%) [Age]	51.8 [19.8]	51.4 [20.4]	50.4 [19.2]	51.2 [12]	54.3 [21.3]
Hb pre-OLT (g/dl)	12.0	12.6	10.2	12.0	10.5
Hb at PTE diagnosis (g/dl)	17.8	17.7	17.6	16.3	18.0
EPO at PTE diagnosis (mU/ml)	14	13	13.8	17.5	38
Creatinine clearance at PTE diagnosis (ml/min)	119	116	101.7	72.6	91
Renal US/TC at PTE diagnosis	Normal	Normal	Normal	Normal	Multiple cysts in both kidneys
Signs and symptoms related to PTE	No	No	No	No	Yes
Gene variants	—	*VHL*(c.552 C/T)	—	*EGLN1*(InsGCC 7x)*	—

EPO: erythropoietin; Hb: hemoglobin; Hct: hematocrit; OLT: orthotopic liver transplantation; PTE: post-liver transplantation erythrocytosis; CT: computed tomography; US: ultrasound.

*The same mutation in the healthy father.

**Table 2:** Comparison between patients affected and patients not affected by post-liver transplantation erythrocytosis.

	Patients with PTE (n = 5)	Patients without PTE (n = 85)	p-value
Male/female (n)	4/1	44/41	0.367
Age at OLT (years)	2.9 [1.3–11.4] (0.8–12.5)	1.5 [0.7–4] (0.2–16.5)	0.172
Age at last follow up (years)	17.5 [13.2–20.6] (12–21.3)	11 [0.7–4] (1.7–24)	0.165
Follow up (years)	12.9 [8.7–17.8] (7.9–20.5)	8.6 [4.7–13.4] (1–20.4)	0.521

Data are presented as median, interquartile range (IQR) and range for continuous not normally distributed variables, and as numbers and proportions for gender. Comparisons are made with Mann-Whitney U test for continuous non parametric variables, and with Fisher exact test for gender. OLT: orthotopic liver transplantation.

**Figure 2:**
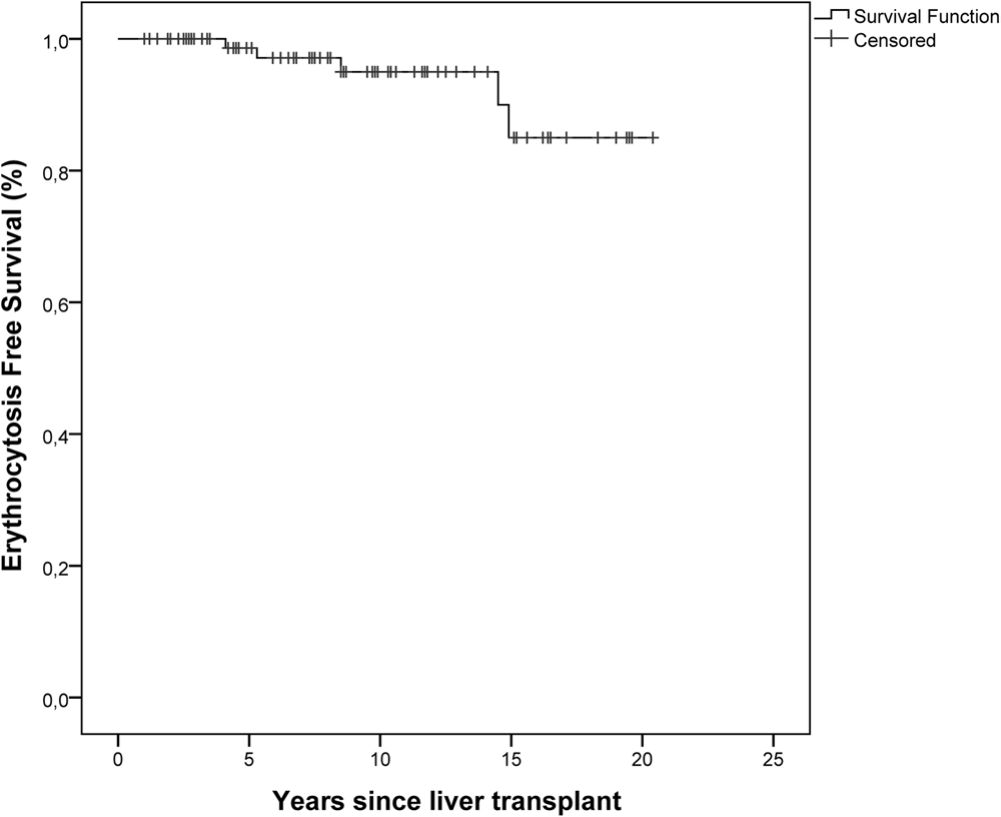
Kaplan-Meier erythocytosis event-free survival curve.

**Figure 3:**
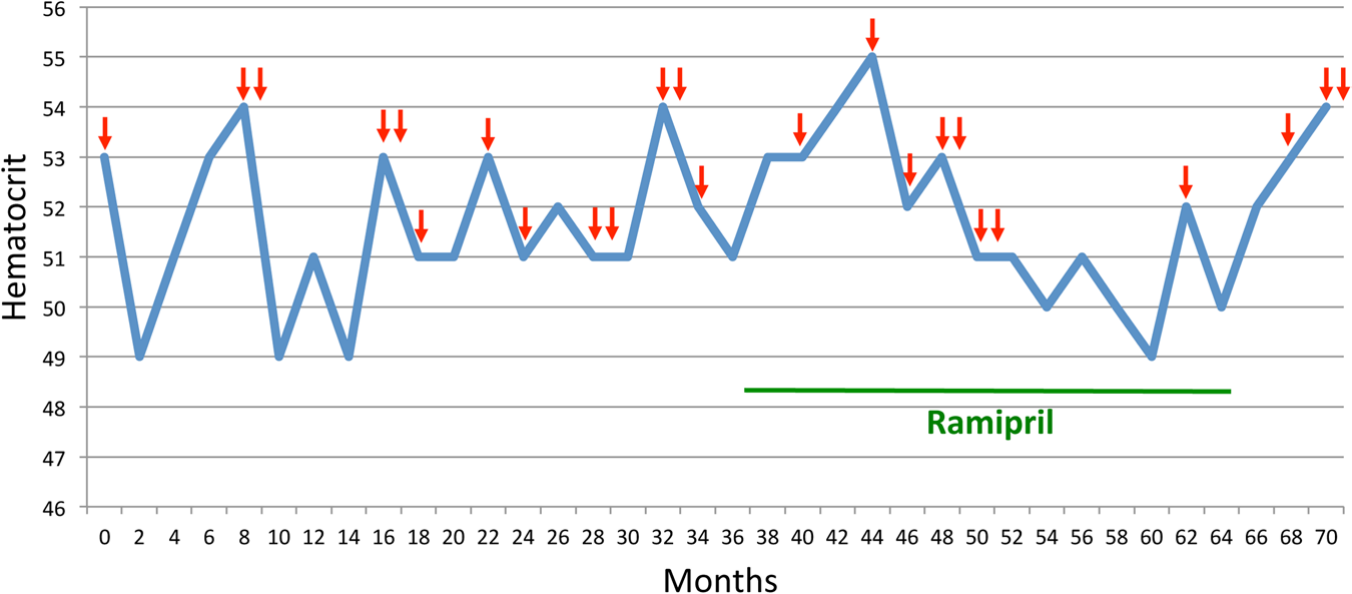
Follow up of patient 5 upon post-liver transplantation erythrocytosis (PTE). Arrows indicate phlebotomy/erythroapheresis sessions.

Erythrocytosis-free survival was 98.6% at 5 years after liver transplantation, 95% at 10 years, and 85% at 15 years (Fig. 2). No death occurred during follow up. Four out of 5 patients developed no clinical signs or complications related to erythrocytosis, and no phlebotomies were required. However, patient 5 developed headache, malaise, dizziness, and hypertension at the age of 15 years (i.e. 14.5 years post-OLT). At diagnosis of erythrocytosis, EPO serum levels were two times the upper limit of normal (ULN) and progressively reached nearly four times ULN (82 mU/ml) over time. Active renin serum levels were normal. The patient developed multiple cysts in both kidneys at 13 years of age; total body CT scan showed no aberrant mass but confirmed the presence of renal cysts. During a 6-year follow up, repeated phlebotomy (n = 10) and erythroapheresis (n = 14) were needed to reduce clinical symptoms. An ACE inhibitor (Ramipril) was added to the treatment plan because of persistence of hypertension and erythrocytosis; response was initially poor, but eighteen months after the start of Ramipril treatment Hct and Hb levels returned to normal, and no further phlebotomy or erythroapheresis were required. An attempted interruption of Ramipril led to hematologic relapse (Fig. 3).

### Gene mutation analysis

All patients with erythrocytosis underwent mutational analysis of genes involved in congenital erythrocytosis (Table 3), according to the protocol adopted at our center. Direct sequencing of the exons and exon-intron boundaries of VHL gene revealed a heterozygous C > T transition at nucleotide 552 in exon 3, causing a synonymous variation in only one patient (Patient 2). This variation does not alter the protein sequence (Leu184Leu). The transition was not found in single nucleotide polymorphism databases^25^ or by DNA sequencing in 200 healthy individuals. In order to evaluate if the VHL C > T transition affects the expression of VHL allele, or results in altered transcripts, we analyzed transcripts for patient 2. We found 2 full-length VHL transcripts as control. We also evaluated the VHL transcript using quantitative real-time PCR. No difference in VHL mRNA levels compared to control was observed (data not shown).

**Table 3:** Genes involved in juvenile erythrocytosis.

OMIM	Category	Gene	Epo level
133100	ECTY1	EPOR	Low
263400	ECTY2	VHL	High
609820	ECTY3	PHD2	Normal
611783	ECTY4	HIF2α	High
617907	ECTY5	EPO	High
617980	ECTY6	HBB	Normal/High
617981	ECTY7	HBA1/2	Normal/High
222800	ECTY8	BPGM	Normal/High
263300	PV	JAK2/TET2/NFE2	Low

ECTY 1–8: familiar erythrocytosis type 1–9; EPOR: erythropoietin receptor; VHL: Von Hippel-Lindau; PHD2: prolyl hydroxylase domain protein 2; HIF2α: hypoxia inducible factor 2α; EPO: erythropoietin; HBB: genes that encode the β globulin chains of hemoglobin; HBA1/2: genes that encode α globin chains of hemoglobin; BPGM: bisphosphoglycerate mutase; JAK2: Janus kinase 2; TET2: Ten-Eleven Translocation 2; NEF2: Nuclear Factor, Erythroid 2.

Direct sequencing of prolyl hydroxylase domain protein 2 gene showed a *de novo* insertion (ins GCC) of a further triplet in the 5′UTR, which normally contains 6 GCC triplet repeats just before the ATG of the gene, causing a frameshift in the affected patient (Patient 4). The insertion was not found in single nucleotide polymorphism databases^25^ or by DNA sequencing in 200 healthy individuals. Genetic analysis of the parents showed the same mutation in the healthy father, thus suggesting the mutation is not pathogenic.

## Discussion

Post-transplant erythrocytosis has usually been described in isolated renal graft and simultaneous kidney-pancreas recipients, and mainly involves adults^5–8,11^. While the pathogenesis of PTE in pediatric OLT is still not completely understood, it is thought to depend on a variety of factors. As in post-kidney transplant erythrocytosis, at least three hormonal systems (erythropoietin, renin-angiotensin system [RAS], and endogenous androgens) are thought to be involved^5^. Indeed, the liver is the site of erythrocyte production from the third to the seventh month of gestation. After birth, erythropoiesis is restricted to the bone marrow from where it can ‘migrate’ (usually to the spleen) in the case of severe anemia. However, there have been anecdotal reports of erythroid precursor cells being detected in a number of human liver grafts in routine biopsies^26^; these findings were limited to adults during the first weeks after OLT and seemed to correlate with graft damage, regenerative activity, and intragraft immune reactions.

Kidney transplant recipients develop erythrocytosis during the first years of follow up^5^ while medical complications of liver transplantation can present either immediately after surgery or several years later. In our study cohort, erythrocytosis was diagnosed 4.1–14.9 years after OLT.

In adult OLT recipients, idiopathic erythrocytosis has been reported in approximately 10% of patients. It occurs within the first two years after liver transplantation in HBV-positive cirrhotic males^13^. Our pediatric cohort presents lower incidence rates of PTE. This is probably related to the different indications for OLT. No children have undergone OLT for HBV infection, while this is reported to be a strong risk factor in adults^13^.

However, patients affected by post-OLT erythrocytosis in our pediatric cohort were predominantly males who were diagnosed in puberty when testosterone levels in males increase to those of male adults. The higher prevalence of erythrocytosis observed in our pediatric cohort is also seen in adult OLT recipients, suggesting a possible role for androgens. Androgens may directly stimulate the erythroid progenitor lines or may increase the production of other erythropoietic factors^5^. Indeed, testosterone-induced erythrocytosis through hepicidin pathway modulation has also been reported^27^.

Male patients in our cohort who had received a liver transplant as children developed erythrocytosis many years after surgery, usually in adolescence (age 15.3–18.8 years); the only female patient in the cohort was diagnosed at a much younger age (8.2 years).

It has been suggested that EPO plays an important part in post-OLT erythrocytosis. In a cohort of 10 pediatric OLT recipients, 6 patients presented a transient increase in EPO level a few days after OLT, and 3 patients required phlebotomy to lower Hb and Hct levels. Interestingly, all patients were anemic and had normal EPO values before liver transplantation, thus suggesting that OLT would have determined an increase in EPO and erythrocytosis^13^.

In agreement with previous reports on post-OLT erythrocytosis^5,8,28^, our only symptomatic patient showed high EPO levels, contrary to expectation from the normally negative feedback loop between Hct and EPO secretion^5^. No subclinical hypoxia was detected by repeated blood gas analyses, and the fact that excessive EPO production was only observed in the patient who developed symptomatic erythrocytosis might suggest it was related to the onset of renal cysts. It is not clear whether renal cysts produce EPO or simply cause local ischemic injury by compressing adjacent renal tissue leading to local renal hypoxia and to increased EPO production^8,15^. The possible association between renal cysts and erythrocytosis is supported by the resolution of the condition after drainage or resection of cysts in some reported cases of secondary policythemia in non-transplanted patients^29,30^.

In a large series of 108 pediatric liver transplant patients, CT scan revealed a high incidence (30%) of post-OLT acquired renal cystic disease^31^. A lower incidence (11%), comparable to our results (8.8%), was observed with US in another series of 235 OLT-children^32^. The different incidence of renal cysts is probably due to the lower sensitivity of US compared with CT scan^31^. Unexpectedly, no mention of erythrocytosis was made in these studies. However, renal cysts and kidney disorders have been associated with abnormal liver function in different diseases^33–35^ and in acquired erythrocytosis^31,32,36^. Therefore, OLT recipients, which represent a particularly fragile patient population, should undergo appropriate evaluation in order to exclude these disorders.

Abnormal erythroid precursor sensitivity to EPO might also be implicated^10,37^, and may partly explain the mechanisms underlying erythrocytosis in clinically asymptomatic patients with normal EPO levels and no renal laboratory or US anomalies.

Interestingly, most renal transplant studies reported that erythrocytosis was more common in male patients^5,7,29^ and in those who received cyclosporine A^5,7^. In our series, erythrocytosis developed in four male patients: three of them were receiving tacrolimus, while the fourth started on cyclosporin A. The possible impact of immunosuppressive agents remains uncertain also because the suggested drug-related effect developed several years after OLT.

In order to identify possible risk factors for post-OLT erythrocytosis, we screened all the genes currently known to be more frequently related to different forms of erythrocytosis^18,23^. This is the first comprehensive and systematic mutational analysis in affected patients. One patient presented genetic mutations compatible with a heterozygous state of VHL transition causing a synonymous variation that does not alter the protein sequence. Moreover, the female patient had a mutated gene involved in the oxygen-sensing pathway. However, this mutation, found also in the healthy father, is not thought to be pathogenic. Therefore, the screened genetic mutations are not involved in the genesis of post-OLT erythrocytosis. Genetic variations in the donor could not be excluded as a possible reason for the negative outcomes of OLTs and for the onset of erythrocytosis in this category of patients. This requires further evaluation.

While post-renal transplantation erythrocytosis has frequently been associated with significant thromboembolic events, and sometimes death^5^, no definitive data about the outcome of post-OLT erythrocytosis in adult patients have been reported. In the Italian cohort of 96 OLT recipients^13^, 11 patients developed erythrocytosis and underwent serial phlebotomy to maintain Hct <49%. There were no reports of cardiovascular events during the observation period; however, 3 out of 11 patients with erythrocytosis (27%) had a history of vascular complications several years after OLT. In adults, erythrocytosis treatment is considered necessary to reduce the potential thromboembolic risk secondary to blood hyperviscosity^5,8,12,37^. Phlebotomy/erythroapheresis is the first-line standard of care^32^. Some drugs, such as angiotensin converting enzyme inhibitors, angiotensin-II receptor antagonist and adenosine receptor antagonist have been reported to reduce high Hct in kidney recipients^5,38^. In particular, it has been demonstrated that Ramipril may be effective in the post-renal trasplant erythrocytosis; low doses normalized Hct in most patients^39^. In our symptomatic patient, phlebotomy/erythroapheresis were well-tolerated and no severe iron deficiency developed. Because of persistence of clinical and laboratory abnormalities, Ramipril was introduced. Although response was initially modest, Hct and Hb levels returned to normal after 18 months of therapy. It is interesting to note that drug discontinuance led to a renewed increase in Hb and Ht values, as has been already described for Enalapril^40^.

In the other patients, due to absence of clinical symptoms and to fluctuations in Hct values, no phlebotomy/erythroapheresis or pharmacological therapy has been started. Pending more definite management criteria^37^, a longer follow up was considered necessary in order to evaluate the course of the erythrocytosis and the need for treatment.

It is worthy of note that, unlike in the post-renal transplant setting, erythrocytosis in our pediatric cohort does not appear to be associated with any adverse symptoms in the majority of cases, or any hitherto increased risk of thrombosis.

In conclusion, erythrocytosis is a complication that occurs several years after OLT in approximately 5% of children, typically during adolescence. The pathogenesis is still not completely understood, but known erythrocytosis-linked genetic factors do not seem to be involved in its development, while male gender, pubertal age, and renal cysts are likely to play a role. Due to the late onset and scarce symptoms, long-term monitoring for erythrocytosis in this patient population should be considered, particularly in adolescence. Epidemiological studies on erythrocytosis in the general pediatric population, and in post OLT patients in particular, are needed to establish whether it is caused by an association between hematologic abnormality and liver transplantation or not. Given the improved survival rates of liver transplant recipients in most centers, greater attention must be paid to rare complications that might develop in the medium-/long-term^41–43^. These are very often related to the immunosuppressive treatment received, although other factors can be involved. These complications may be mis-diagnosed and not considered as being transplant related. However, since, in general, patients are now living longer after OLT, the number of late complications is likely to increase. In addition, risk factors for PTE have to be carefully evaluated for the potential co-morbidity and fatal outcome of hematologic/vascular complications, such as hypertension, thrombosis, and metabolic imbalance.
